# Heterologous Expression and Functional Verification of Extracellular Carbonic Anhydrases in *Bacillus safensis yw6* from Mariana Trench

**DOI:** 10.3390/molecules29245911

**Published:** 2024-12-14

**Authors:** Xinyu Wang, Pengna Wang, Hancheng Zhao, Yingying He, Changfeng Qu, Jinlai Miao

**Affiliations:** 1 Marine Natural Products Research and Development Key Laboratory of Qingdao, First Institute of Oceanography, Ministry of Natural Resources, Qingdao 266061, China; wangxinyu@fio.org.cn (X.W.); yufei2468@126.com (P.W.); zhaohc@fio.org.cn (H.Z.); yingyinghe@fio.org.cn (Y.H.); 2 Laboratory for Marine Drugs and Bioproducts, Qingdao Marine Science and Technology Center, Qingdao 266237, China

**Keywords:** extracellular carbonic anhydrase, *Bacillus schafferii*, bioinformatics analysis, heterologous expression

## Abstract

The exploration and exploitation of deep-sea microbial resources is of great scientific value for understanding biological evolution under extreme conditions. Deep-sea microorganisms are critical in the ocean carbon cycle, and marine heterotrophic microorganisms secrete extracellular carbonic anhydrase (CA) to fix inorganic carbon, an important process in climate regulation. Extracellular CA provides a green method for fixing carbon dioxide into stable minerals containing Ca^2+^. However, studies on extracellular CA in deep-sea microorganisms are limited. In this study, *Bacillus safensis yw6* was isolated from Mariana Trench sediments and three candidate extracellular CA genes (*β-ca*1, *β-ca*2, and *γ-ca*) were identified by whole genome sequencing. Bioinformatics analyses showed that these CAs have different structural compositions, with the β-CA having α-helix and random coiling, whereas the γ-CA has more random coiling and stretched strands. Heterologous expression in E. coli BL21 (DE3) showed that β-CA2 had the highest enzyme activity, followed by γ-CA and β-CA1. Field emission scanning electron microscopy (FESEM) observations showed that the engineered strains with *β-ca*2 genes produced deposits that were like those from natural sources. This finding not only provides new perspectives for the utilization of deep-sea microbial resources, but also provides an important scientific basis for the molecular mechanisms of extracellular CAs of deep-sea microbes.

## 1. Introduction

To slow down global warming, various methods have been explored to fix carbon dioxide in the atmosphere. Traditional physic-chemical methods are effective but are often associated with significant energy consumption and may not be environmentally friendly [1]. In contrast, plants fix CO_2_ in a more natural way through photosynthesis, however, the limited growth range of plants restricts their potential for carbon sequestration. Microorganisms, as a widespread group of organisms on earth, have become a hotspot for carbon sequestration research due to their unique growth and adaptation characteristics [2]. The great ability of microorganisms to reproduce and their strong adaptability to their environment allows them to sequester carbon in places where many plants cannot grow [3]. In addition, microbial carbon sequestration usually does not require additional energy input, which makes microbial carbon sequestration a more energy-efficient and environmentally friendly way of carbon sequestration [4].

The fixation of inorganic carbon by the oceans has become one of the key instruments of climate regulation. The oceans have absorbed about 25% of anthropogenic carbon dioxide emissions since the beginning of the industrial revolution. [5]. The deep-sea provides a rich and diverse ecological environment for the evolution and reproduction of microorganisms [6]. Many microorganisms can survive in extreme deep-sea environments, such as low and high temperatures, and they have developed new biochemical mechanisms and signaling systems, highly efficient enzymes, secondary metabolites, and unique genetic traits that are distinct from those of terrestrial organisms during their evolution [7]. In marine sediments, bacteria play an important role in ocean mineralization and have a significant impact on the marine carbon cycle [8]. Therefore, the exploitation of microbial resources in the deep-sea environment is important for CO_2_ fixation. Carbon fixing microorganisms in the ocean include several types of photosynthetic autotrophic microorganisms, chemically energetic autotrophic microorganisms, and heterotrophic microorganisms [9]. Among them, heterotrophic microorganisms secrete extracellular carbonic anhydrase (CA), which catalyzes the reversible reaction of CO_2_ and HCO^3−^ to produce stable minerals in the presence of Ca^2+^ [10]. Carbonic anhydrase can increase the enzymatic reaction rate by seven orders of magnitude, providing an efficient and environmentally friendly enzymatic method for carbon dioxide (CO_2_) fixation [11].

Carbonic anhydrases are a class of metal ion-containing enzymes whose main function is to reversibly catalyze the CO_2_ hydration reaction to form metal-bound HCO^3−^, thereby fixing CO_2_ [12]. CAs are ubiquitous in microorganisms, and bacterially encoded carbonic anhydrases belong to three distinct genetic families, namely the α-, β-, and γ- classes, where β- and γ- predominate, and α- is present only relatively rarely in bacteria [13,14]. CA has attracted the attention of scientists because of its high efficiency and specificity, while its catalytic CO_2_ conversion has received the most attention, and the research direction has gradually moved from theory to practical application, with specific applications involving the following aspects:

In the field of ecological conservation, biological carbon sequestration using carbonic anhydrase is regarded as a very cost-effective strategy to mitigate global warming. Carbonic anhydrase not only enhances the photosynthesis of algae by intervening in the CO_2_ concentration mechanism to achieve carbon fixation [14], but also converts unstable carbon in nature into stable forms by means of the biomineralization process [15,16]. This biomineralization pathway not only contributes to the reduction in atmospheric CO_2_ concentration, but also effectively mitigates the pollution of the environment with metal ions such as Mg^2^⁺, Mn^2^⁺, Fe^2^⁺, Ca^2^⁺, and Co^2^⁺ [17,18]. In terms of industrial applications, aqueous solutions of carbonic anhydrase can be used in industrial carbon dioxide capture and storage processes as an alternative to amine-based solutions, which are energy-demanding and environmentally unfavorable [19]. With the help of bacterial-induced calcite precipitation reaction, this technology can be effective in dust suppression, the remediation of contaminated soils, the calcium ion sequestration of PCBs, and the remediation of soils and buildings [20]. In addition, carbonic anhydrase has been used to drive a photocatalytic hydrogen production system that is less expensive and avoids the need to use precious metals and organic donors [21]. In the field of medicine, the inhibition of carbonic anhydrase (CA) has been developed for use as diuretics, antiepileptic drugs, glaucoma therapeutic agents, anti-obesity drugs, and antitumor agents [22]. Studies have shown that osteosclerosis accompanied by renal tubular acidosis and cerebral calcification syndrome are caused by type II carbonic anhydrase deficiency, and in-depth studies of carbonic anhydrase have helped us understand the pathogenesis of these disorders and to explore appropriate therapeutic options [23]. In addition, carbonic anhydrase has been used in the treatment of cancer [24]. In agriculture, the introduction of the carbon concentration mechanism of algae into the chloroplasts of terrestrial C_3_ plants, such as tobacco, is expected to enhance crop productivity and yield [25]. In addition, β-type carbonic anhydrase is regarded as a highly promising targeted insecticide in agriculture and animal husbandry [26,27].

In this study, we used bioinformatics techniques to analyze the whole genome of a deep-sea-derived strain of *B. safensis yw6* to identify candidate carbonic anhydrase genes and to predict the structure of the proteins they encode. In addition, we achieved the heterologous expression of each of these three CA genes in Escherichia coli BL21 (DE3) and performed enzyme activity assays using the CO_2_ hydration method.

## 2. Results

### 2.1. Bioinformatic Analysis

The results showed that the ORF lengths of *β-ca*1, *β-ca*2, and *γ-ca* genes were 579 bp, 597 bp, and 576 bp, encoding 192, 198, and 191 amino acid sequences, respectively. Subsequently, the physicochemical properties of the three proteins, β-CA1, β-CA2, and γ-CA, were analyzed as follows. The predicted isoelectric points (pI) of β-CA1, β-CA2, and γ-CA were 6.26, 6.71, and 7.02, respectively, and the instability indexes were 37.14, 41.43, and 46.42, suggesting that these three proteins tended to exist stably in the in vitro environment. The aliphatic indexes of β-CA1, β-CA2, and γ-CA were 100.69, 102.37, and 101.99, respectively, and the estimated total atomic numbers were 3280, 3089, and 2986, respectively, with the relative molecular masses of about 22.99 kDa, 21.67 kDa, and 20.99 kDa and the molecular formulae were C_1035_H_1666_N_266_O_304_S_9_, C_965_H_1572_N_260_O_281_S_11_, C_936_H_1507_N_261_O_278_S_4_. All three proteins were determined to be hydrophilic proteins by ExPASy Scale program analysis, with average hydrophilicity coefficients (GRAVY) of −0.188, −0.060, and −0.107, respectively, which further supported their hydrophilicity (Table 1).

**Figure 1:**
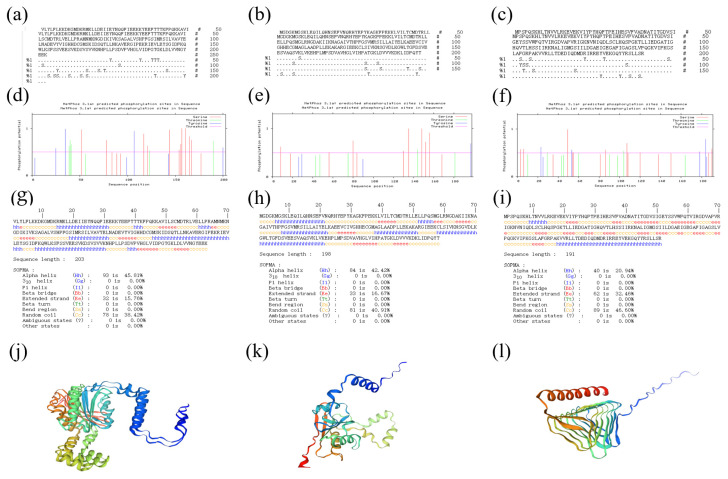
Prediction plot of phosphate sites in amino acid sequence of β-CA1 (**a**), β-CA2 (**b**) and γ-CA (**c**); Predictive analysis of sequence phosphate sites of β-CA1 (**d**), β-CA2 (**e**), and γ-CA (**f**); Secondary structure and distribution of β-CA1 (**g**), β-CA2 (**h**), and γ-CA (**i**); Prediction of the tertiary structure of β-CA1 (**j**), β-CA2 (**k**), and γ-CA (**l**).

The results of phosphorylation site prediction showed that 20 potential phosphorylation sites were predicted to exist in the amino acid sequence of β-CA1, specifically including four tyrosine (Y) sites (Y_25_, Y_35_, Y_107_, Y_200_), five threonine (T) sites (T_39_, T_40_, T_41_, T_123_, T_190_), and 11 serine (S) sites (S_52_, S_78_, S_117_, S_121_, S_144_, S_154_, S_156_, S_157_, S_161_, S_165_, S_167_). For β-CA2, a total of 12 potential phosphorylation sites were predicted in its amino acid sequence, of which serine (S) accounted for 9 (S_8_, S_56_, S_80_, S_84_, S_130_, S_135_, S_148_, S_152_, S_156_), threonine (T) for 2 (T_144_, T_181_), and tyrosine (Y) for only 1 (Y_197_). A total of 15 potential phosphorylation sites were predicted in the amino acid sequence of γ-CA, which were distributed as follows: 10 serine (S) (S_3_, S_6_, S_49_, S_54_, S_55_, S_86_, S_107_, S_120_, S_187_, S_190_), 4 tyrosine (Y) (Y_23_, Y_53_, Y_178_, Y_184_), and 2 threonine (T) (T_29_, T_104_). These predictions provide key information for further investigation of the effects of phosphorylation on protein conformation and function. The results of the secondary structure prediction of the three proteins showed that the secondary structure of β-CA1 was mainly composed of α-helices (Hh, 45.81%), irregular coils (Cc, 38.42%), and extended chains (Ee, 15.76%). For β-CA2, its secondary structure similarly contains α-helices (Hh, 42.42%), irregular curls (Cc, 40.91%) and extended chains (Ee, 16.67%). As for γ-CA, its secondary structure consists of α-helix (Hh, 20.94%), irregular curl (Cc, 46.60%), and extended chain (Ee, 32.46%). The results of the tertiary structure prediction of the three proteins showed that the predicted tertiary structures of these proteins coincided with the conformations and ratios of their respective secondary structures, verifying the accuracy and reliability of the predictions (Figure 1).

**Figure 2:**
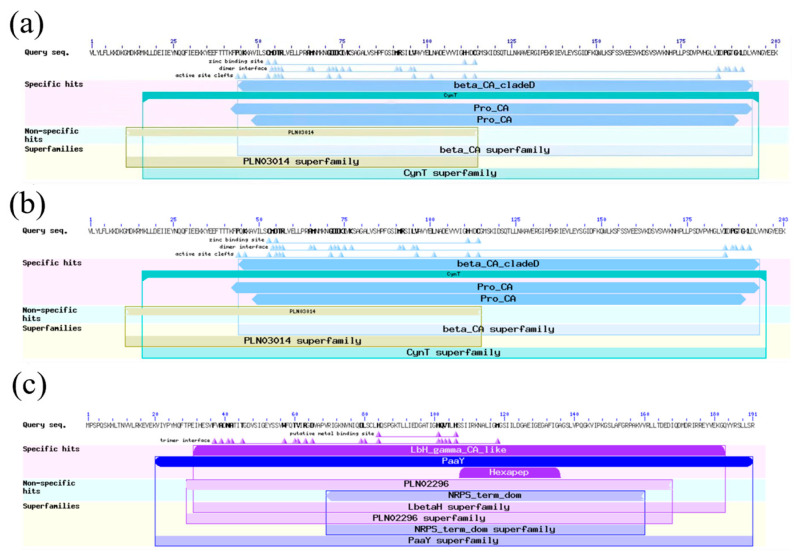
Protein functional domain prediction of β-CA1 (**a**), β-CA2 (**b**), γ-CA (**c**).

Using the NCBI BLAST tool, we identified the protein functional domains corresponding to the β-CA1, β-CA2, and γ-CA amino acid sequences. Specifically, the β-CA1 protein contains the β-CA protein structural domain, classified in the β-carbonic anhydrase family and the CynT superfamily, and its function involves inorganic ion transport and metabolic processes. The structural features of this protein include four Zn^2^⁺ binding sites, 15 dimeric interaction interfaces, and 12 active activation sites. Similarly, the β-CA2 protein also contains the β-CA protein structural domain and belongs to the β carbonic anhydrase family and the CynT superfamily, which are involved in the transport and metabolism of inorganic ions. Its structural features are demonstrated as 4 Zn^2^⁺ binding sites, 14 dimer interfaces, and 12 active sites, and the functional domains of β-CA2 are highly like those of β-CA1. As for the γ-CA protein, it contains the γ-CA protein domain, the PaaY domain, and the carbonate dehydratase domain, and belongs to the γ-carbonic anhydrase family. γ-CA is structurally characterized by 3 Zn^2^⁺-binding sites and 18 dimeric interfaces (Figure 2).

**Figure 3:**
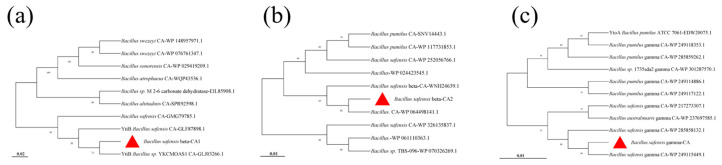
Phylogenetic tree of β-CA1 (**a**), β-CA2 (**b**), and γ-CA (**c**). The red triangles represent the proteins β-CA1, β-CA2 and γ-CA derived from *Bacillus safensis*, respectively.

The results of the evolutionary tree analysis for β-CA1 showed that it has the closest affinity to the *Bacillus* sp. *YKCMOAS1 CA-GLJ03266.1* derived carbonic anhydrase, with 73% Bootstrap support. For β-CA2, the evolutionary tree revealed that it has the closest affinity to carbonic anhydrase represented by the sequence *Bacillus. CA-WP 064498141.1*, with 83% Bootstrap support. The evolutionary tree analysis of γ-CA revealed that it was most closely related to sequence *Bacillus safensis gamma CA-WP 249115449.1*, which belongs to the same genus *Bacillus safensis,* with a Bootstrap support of 84% (Figure 3).

### 2.2. The ca Gene Extraction and Detection

**Figure 4:**
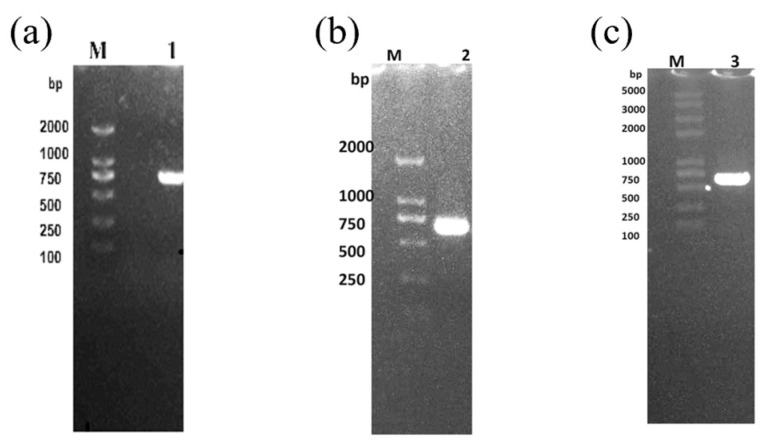
Plot of the results of electrophoretic detection of *β-ca*1 (**a**), *β-ca*2 (**b**) and *γ-ca* (**c**) genes amplification products. Lane M: DNA maker, Lane 1: *β-ca*1, Lane 2: *β-ca*2, Lane 3: *γ-ca*.

The electrophoresis results were observed by gel imaging system (Figure 4), and the length of the amplification products of the three ca genes was about 600 base pairs (bp), which was in line with the theoretical expectation, indicating that the amplification products could be used for subsequent cloning and ligation as well as transformation experiments.

### 2.3. The ca Genes Cloning and Expression

**Figure 5:**
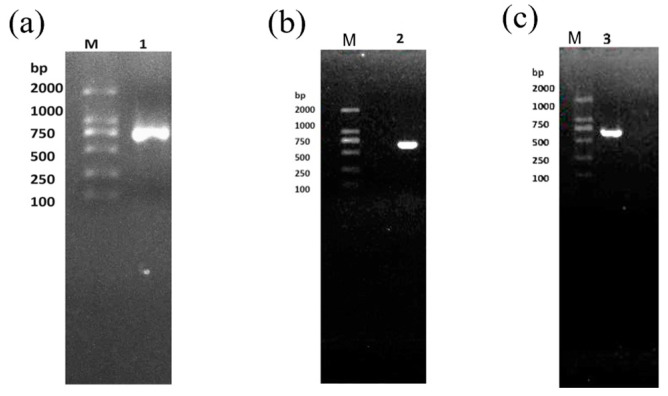
Electrophoretic detection of PCR products from recombinant plasmid *β-ca*1 (**a**), *β-ca*2 (**b**) and *γ-ca* (**c**). Lane M: DNA maker, Lane 1: *β-ca*1, Lane 2: *β-ca*2, Lane 3: *γ-ca*.

The results of the electrophoresis assay are shown in Figure 5, where a band size of approximately 750 bp was observed, which is consistent with the expected size of the target band.

### 2.4. Expression and Purification of Extracellular CA Proteins

**Figure 6:**
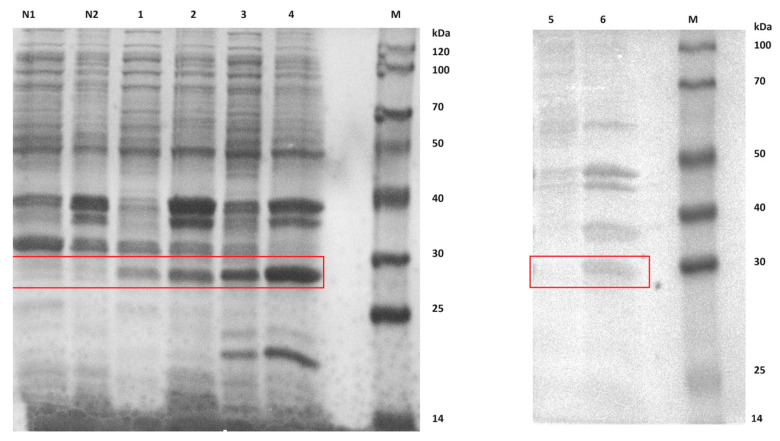
SDS-PAGE electrophoresis detection of the induced CA protein (The exact location of the target protein is circled in red box). M: Protein Marker, N1: blank supernatant, N2: blank inclusion, 1: β-CA2 supernatant, 2: β-CA2 inclusion, 3: γ-CA supernatant, 4: γ-CA inclusion, 5: β-CA1 inclusion, 6: β-CA1 supernatant.

Comparative analysis of the differences in protein expression between recombinant strains introduced with the *ca* gene and control strains introduced with blank plasmids. According to the electrophoresis results (Figure 6), we can observe protein bands for β-CA1, β-CA2, and γ-CA. According to theoretical calculations, the relative molecular mass of β-CA1 should be 22.99 kDa, that of β-CA2 is 21.67 kDa, and that of γ-CA is 20.99 kDa. However, in the electrophoresis profile, the apparent molecular weights of these CA proteins did not agree with the theoretical values, and they all showed molecular weights in the range of 25–30 kDa (marked by red boxes in the figure). Notably, all three proteins were expressed in the supernatant, suggesting their potential for further use in enzyme activity assays.

**Figure 7:**
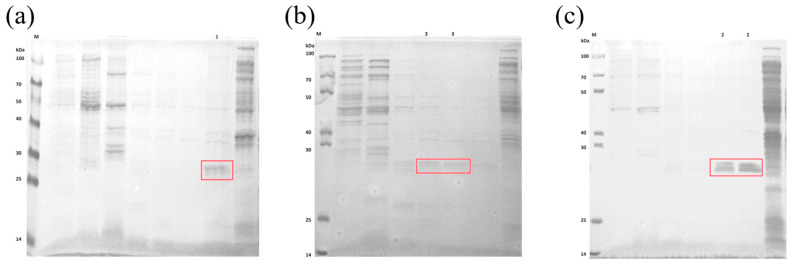
Purified protein detected by SDS-PAGE electrophoresis (The exact location of the target protein is circled in red box). M: marker;1: purified protein; (**a**) Purified protein detected by SDS-PAGE electrophoresis of β-CA1; (**b**) Purified protein detected by SDS-PAGE electrophoresis of β-CA2; and (**c**) Purified protein detected by SDS-PAGE electrophoresis of γ-CA.

After the purification process on the Ni-IDA column, we performed the SDS-PAGE electrophoretic analysis of the CA proteins in the three supernatants with the aim of exploring their binding properties with different concentrations of the imidazole eluent. Specifically, β-CA1 protein was efficiently eluted at an imidazole concentration of 500 mM; whereas the β-CA2 protein was eluted at both imidazole concentrations of 200 mM and 500 mM; in addition, γ-CA protein was eluted at relatively low imidazole concentrations (80 mM and 120 mM) (Figure 7). It is noteworthy that β-CA1 and γ-CA required somewhat lower concentrations of eluting imidazole compared to β-CA2.

### 2.5. Determination of Extracellular CAs Activity of Engineering Bacteria

We assessed the CA enzyme activity using the CO_2_ hydration method, and the resulting protein concentration and enzyme activity data are detailed in Table 2. The results showed that the γ-CA protein had the highest expression prior to purification, whereas the β-CA2 protein was harvested more significantly compared to the other two types of CAs during the purification process. The enzyme activity assay further indicated that β-CA2 was characterized by high activity (before purification, its enzyme activity was as high as 4.92 U with a specific activity of 0.641 U/mg; after purification, although the enzyme activity was reduced to 0.081 U), and its specific activity was elevated to 0.409 U/mg.

**Figure 8:**
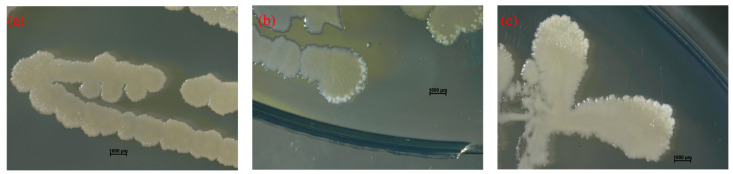
Surface morphology of strains in solid M1 + CaCl_2_ medium. (**a**) Surface morphology of blank control; (**b**) Surface morphology of wild strains; and (**c**) Surface morphology of *β-ca*2 gene-containing engineered strains.

It was rigorously verified that the engineered bacteria carrying β-CA2 showed the highest carbonic anhydrase activity. The engineered bacteria and wild strains were inoculated on the surface of M1 solid medium coated with 34 mM calcium chloride (CaCl_2_). The observation of the surface morphology of the colonies revealed white deposits on the edges of both wild and engineered strains (Figure 8).

**Figure 9:**
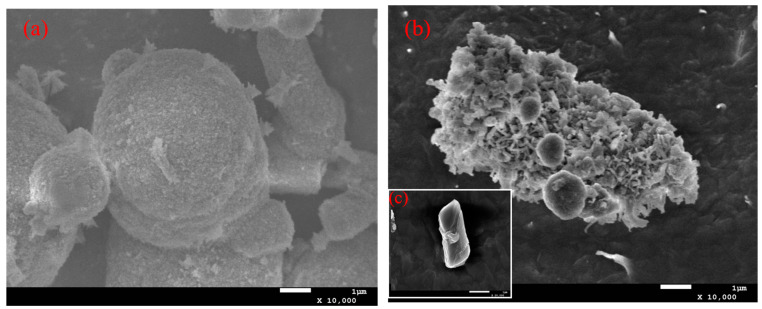
Electron microscope scans of white deposits produced by wild strains (**a**) and *β-ca*2 gene-containing engineered strains (**b**); and (**c**) local magnification of the figure in (**b**) by a factor of 10,000.

White deposits produced by colonies were observed by field emission scanning electron microscopy (FE-SEM). Observations showed that these minerals exhibit a variety of morphologies, including spherical, rhombic, and irregular shapes (Figure 9). It is particularly noteworthy that the crystal morphology demonstrated in Figure 9c is very similar to the rhombohedral morphology of calcite, which strongly suggests that the engineered strains with the introduction of the *β-ca*2 gene have higher stability in the formation of mineral precipitates as compared to the wild strains [28].

## 3. Discussion

Global warming has become a central topic in the scientific community, in the field of environmental protection and in global economic discussions. The oceans, as a central element in the regulation of atmospheric CO_2_ concentrations, play a decisive role in the long-term evolution of the Earth’s climate over thousands and tens of thousands of years. Marine microorganisms, as the main drivers of ocean biogeochemical cycles, play an indispensable role in maintaining the balance of marine ecosystems and building carbon reservoirs [29]. Ocean carbon sequestration technology is based on microbial carbon pumping (MCP), which endeavors to deeply bury particulate organic carbon and insoluble carbonates in deep-sea or seafloor sediments to achieve long-term or even permanent carbon sequestration [30]. MCP involves a series of complex processes that realize the supply, consumption, and storage of carbon in the ocean [31]. At the same time, the functioning of the carbonate pump heavily relies on the production of calcium carbonate (PIC), i.e., particulate inorganic carbon, synthesized by calcifying microorganisms, and its delivery to deep-sea regions [32]. Most importantly, the long-term stability of geologically sequestered CO_2_ depends on the catalytic efficacy of microbial CAs. Previous studies have shown that CAs are widely present in vertebrate erythrocytes as well as in a wide range of plant and animal tissues [33], and the function of the CA family in terrestrial plants and animals has been well documented [34]. Although photosynthesis in plants has received much attention in terms of carbon fixation, its growth range is relatively limited. In contrast, microorganisms have become the preferred biomaterials for carbon fixation studies due to their rapid growth rate and excellent environmental adaptability [35]. Extracellular CAs secreted by heterotrophic microorganisms are one of the fastest enzymes known for enzymatic reactions and are capable of dramatically increasing the reaction rate up to seven orders of magnitude, providing an efficient and environmentally friendly enzymatic strategy for CO_2_ capture and conversion.

In this paper, we report three *ca* genes, named *β-ca*1, *β-ca*2, and *γ-ca*, successfully obtained from the *B. safensis yw6* strain. To deeply investigate the function and mechanism of action of the proteins encoded by these genes, we used a bioinformatics approach to analyze and predict the structure of the amino acid sequences they encode. The results showed that the protein encoded by *β-ca*1 gene contained 192 amino acid residues with a relative molecular mass of 22.99 kDa, which was hydrophilic and stable; the protein encoded by the *β-ca*2 gene contained 198 amino acid residues with a relative molecular mass of 21.67 kDa, which was also hydrophilic but unstable; the protein encoded by the *γ-ca1* gene consisted of 191 amino acid residues with a relative molecular mass of 20.99 kDa, which was also hydrophilic but unstable. The protein encoded by the *γ-ca*1 gene consists of 191 amino acid residues with a relative molecular mass of 20.99 kDa and is also hydrophilic but unstable. It is noteworthy that all three extracellular CAs are zinc enzymes, and their active sites are highly conserved. In the secondary structure prediction, we find that all three CAs consist of structural elements such as α-helices, irregular coiling, and extended chains. Specifically, β-CA1 and β-CA2 have α-helix and irregular curls as their main structural features, and contains a certain number of extensional chains, while γ-CA has a higher proportion of irregular curls and extensional chains than α-helix. These structural features provide important clues to further reveal the functions and mechanisms of these CAs.

Immediately after that, we successfully cloned and expressed three extracellular *ca* genes from *B. safensis yw6* strain. Meanwhile, we extracted and purified the expressed proteins. To quantify the activity level of the CAs enzyme, we used the CO_2_ hydration method for the assay. The results of the enzyme activity assay clearly showed that the three CA enzymes, in descending order of viability, were β-CA2, γ-CA, and β-CA1.

Multiple CA isoforms are implicated in a range of diseases, including cancer [36], which is one of the key factors that led us to choose to focus our research on CAs. After exhaustive prediction and parsing, we have now confirmed that the *B. safensis yw6* strain contains three *ca* genes. With the help of DNA recombination technology, we successfully expressed CA in a heterologous system and implemented the validation of its hydratase activity. This research result provides a valuable reference for the in-depth development and effective use of deep-sea mineralized biological resources. In addition, while most of the bacteria obtained for calcification experiments and secreting extracellular carbonic anhydrase are from caves and soils, *B. safensis yw6* studied in this thesis is the first *Bacillus safensis* with high carbon sequestration capacity obtained from an extreme environment in the deep sea (Mariana Trench). This is one of the important reasons why we are highly interested in it.

## 4. Materials and Methods

### 4.1. The Origin of the B. safensis yw6

The samples were collected from the Mariana Trench (position: 142°30′22.4664″ E, 10°38′5.3374″ N; water depth: 5150 m), screened from sediments, and preserved in the Marine Microbial Germplasm Repository of the Centre for Research on Marine Biological Resources and Environment, the First Institute of Oceanography, Ministry of Natural Resources.

### 4.2. Bioinformatic Analysis

After obtaining the genomic data of *B. safensis yw6*. The CAs candidate genes in *B. safensis yw6* were screened by comparing them with the sequences of ca genes and CA proteins published in the NCBI database. And on this basis, detailed analysis was carried out by function prediction, sequence comparison, and molecular evolution. Predicting Open Reading Frames (ORFs) in the NCBI Open Reading Frame Finder; the physicochemical property analysis of CAs proteins using the ProtPram tool of the Expert Protein Analysis System database; CA protein hydrophilicity was predicted using the ExPASyScale program; CAs phosphosites were predicted using the NetPhos-3.1 server; CA proteins structural domain analyses were performed at the NCBI website; and the secondary structure of CAs proteins was predicted by the SOPMA protein secondary structure prediction website. The tertiary structure of CA proteins was predicted by homology modeling using SWISS-MODEL online software (https://swissmodel.expasy.org/, accessed on 10 November 2024); multiple sequence comparison analysis of CAs proteins sequences was carried out using DNAMAN9 software; homologous sequences of three CAs proteins were collected by comparison using the protein BLAST program on the NCBI website; and a phylogenetic tree was constructed using MEGA11.

### 4.3. The ca Gene Extraction and Detection

The operating instructions of the DNA extraction kit were followed to extract the genomic DNA from the *B. safensis yw6* strain, and nucleic acid electrophoresis was executed on the extracted genomic DNA. Primers targeting three *ca* genes were designed using Primer Premier 5 software (Table 3). After *B. safensis yw6* was cultured to the logarithmic growth stage, DNA extraction was performed according to the instructions of the kit. Subsequently, the extracted DNA was amplified with the designed primers as templates. The reagent ratios and procedure settings in the 2× TransStart FastPfu Fly PCR SuperMix kit (Transgen Biotech, Beijing, China) instructions were strictly followed.

The amplification products were tested by agarose gel electrophoresis to see whether they matched the size of the target gene. The remaining PCR products were sent to the Sequencing Department of Sangon Biotech Co. (Shanghai, China).

### 4.4. The ca Genes Cloning and Expression

Use the pEASY-Blunt E1 Expression Kit (TransGen Biotech Co., Ltd., Beijing, China) and follow the instructions. The purified *ca* gene was ligated to the Blunt E1 vector and introduced together into the recipient cell, Trans-T1. After the screening of the positive clonal receptor cells, RCR amplification and agarose gel electrophoresis were performed to test the amplification products for size with respect to the target gene. The remaining PCR products were sent to Sangon Biotech Co. for sequencing and purification. ca-Blunt E1 expression vector was sequenced and purified by Sangon Biotech Co. and introduced into expression recipient cells Transetta (DE3). Expression receptor cells Transetta (DE3) were recovered in LB liquid medium. Plates were then coated and inverted for 12 h to screen for positive strains. Positive strains were stored in glycerol at −80 °C in a refrigerator.

### 4.5. Expression and Purification of Extracellular CA Proteins

Blunt E1 Expression Vector carrying the ca gene and a blank control were inoculated into LB liquid medium containing Ampicillin (Amp). After the activated engineered strains were cultured until they reached logarithmic growth phase, IPTG solution was added to the culture system and their expression was induced in an ultra-clean bench for about 30 h. Subsequently, cell precipitates were collected by centrifugation operation and resuspended using phosphate buffer solution (PBS). Next, the resuspended cells were crushed using an ultrasonic cell crusher. The crushed mixture was centrifuged again, separated to obtain the supernatant, and 2 mL of inclusion body lysate was added to it to dissolve the remaining precipitate. Finally, the extracted protein samples were stored frozen at −20 °C for subsequent analysis and determination.

Purify the CAs proteins according to the instructions for the HisTrap HP Histidine Affinity Chromatography Column. The effluent from the purification process will be collected and SDS-PAGE will be applied to confirm the presence of the target protein. In addition, protein concentration will be determined according to the instructions of the BCA Protein Concentration Assay Kit (Solarbio Science & Technology Co., Bingjing, China).

### 4.6. Determination of Extracellular CAs Activity of Engineering Bacteria

The viability of extracellular CAs was detected using the hydropathic method. The three engineered colonies were activated and cultured to the logarithmic growth stage, and the expanded culture was continued until the OD600 value reached 1000. The supernatant was centrifuged, the supernatant was thoroughly mixed with 20 mM Tris-HCl buffer (pH = 8.0), and the initial pH value of the mixture was determined. Next, saturated aqueous CO_2_ was rapidly added to the mixture in an ice-water bath while a pH meter and stopwatch were activated to record the time it took for the pH to decrease from 8.3 to 6.3. To ensure the accuracy of the data, the process was repeated three times and averaged. Finally, the relative activity of extracellular carbonic anhydrase of strain yw6 was calculated according to the equation U = (t_0_ − t)/t.

Subsequently, the strains were inoculated and cultured on M1 medium coated with 34 mM calcium chloride (CaCl_2_) solution. We carefully observed the phenomenon of mineral precipitation formed on the surface of the bacterial colonies using a stereomicroscope and tested their calcification ability according to the literature [37]. The mineral precipitates on the surfaces of the colonies were then collected, gently rinsed with purified water to remove the attached bacteria, dried and sprayed with gold dust to enhance the observation. A field emission scanning electron microscope (FE-SEM) was used to observe the surface morphology of the treated minerals at high resolution, with the following parameters set: the working distance was set to 0.8 nm, the accelerating voltage was adjusted to 15 kV, and it was ensured that the maximum beam current was not more than 2 µA.

## 5. Conclusions

Studies have shown that extracellular CAs plays a significant role in facilitating biomineralization: in this study, for the first time, *B. safensis yw6*, a bacterium with high carbon sequestration capacity, was obtained from an extreme deep-sea environment (Mariana Trench mud samples), and was bioinformatically analyzed to elucidate the structural features and functional properties of its three CA enzymes at the molecular and protein levels. In addition to this, the production of extracellular CAs in the marine environment is limited by specific conditions and low yield. Therefore, in this study, the *ca* gene was introduced into the recipient cells and successfully expressed, which is important to achieve the production of carbonic anhydrase in the common environment. More notably, if this carbonic anhydrase needs to be produced in large quantities, the optimal expression conditions of the *ca* gene need to be mapped out. Therefore, the subsequent work should focus on exploring the optimal induction conditions for efficient CA expression, screening several factors that may have a significant effect on CA expression, and conducting one-way experiments and response surface analyses to support the full use of carbonic anhydrase secreted by deep-sea microorganisms in the future.

## Figures and Tables

**Table 1:** Statistical table of amino acid composition of extracellular CAs.

	β-CA1		β-CA2		γ-CA	
Amino Acid	Number	Percentage	Number	Percentage	Number	Percentage
Ala (A)	7	3.4%	14	7.1%	10	5.2%
Arg (R)	6	3.0%	5	2.5%	11	5.8%
Asn (N)	7	3.4%	5	2.5%	5	2.6%
Asp (D)	13	6.4%	10	5.1%	10	5.2%
Cys (C)	2	1.0%	4	2.0%	1	0.5%
Gln (Q)	5	2.5%	6	3.0%	9	4.7%
Glu (E)	17	8.4%	15	7.6%	11	5.8%
Gly (G)	12	5.9%	17	8.6%	18	9.4%
His (H)	5	2.5%	8	4.0%	6	3.1%
Ile (I)	14	6.9%	12	6.1%	19	9.9%
Leu (L)	21	10.3%	23	11.6%	15	7.9%
Lys (K)	22	10.8%	19	9.6%	10	5.2%
Met (M)	7	3.4%	7	3.5%	3	1.6%
Phe (F)	7	3.4%	4	2.0%	5	2.6%
Pro (P)	8	3.9%	9	4.5%	9	4.7%
Ser (S)	15	7.4%	11	5.6%	15	7.9%
Thr (T)	6	3.0%	6	3.0%	9	4.7%
Trp (W)	1	0.5%	1	0.5%	1	0.5%
Tyr (Y)	7	3.4%	4	2.0%	6	3.1%
Val (V)	21	10.3%	18	9.1%	18	9.4%

**Table 2:** CAs concentration and activity.

Type	Concentration (mg/mL)	Enzyme Activity/U	Vitality Contest (U/mg)
β-CA1	4.724	0.077	0.326
β-CA2	4.303	0.138	0.641
γ-CA	5.647	0.121	0.426
Purified β-CA1	0.85	0.026	0.306
Purified β-CA2	1.98	0.081	0.409
Purified γ-CA	0.83	0.026	0.313

**Table 3:** Primers are used for CA gene cloning.

Name	Primer Nucleic Acid Sequence
*β-ca*1 forward primer	GAAATTTCTTACATTGTGCTATATTTAT
*β-ca*1 reverse primer	TTACTTTTCTTCATATCCGTTGACAA
*β-ca*2 forward primer	ATGGGAGATGGCAAAATGGGAT
*β-ca*2 reverse primer	TTAGGTATACTGCGGGTCAATC
*γ-ca* forward primer	ATGCCAAGTCCACAAAGTAAACAC
*γ-ca* reverse primer	TTAACGAGATAAAAGGGAACGATAG

## Data Availability

The raw data supporting the conclusions of this article will be made available by the authors upon request.
